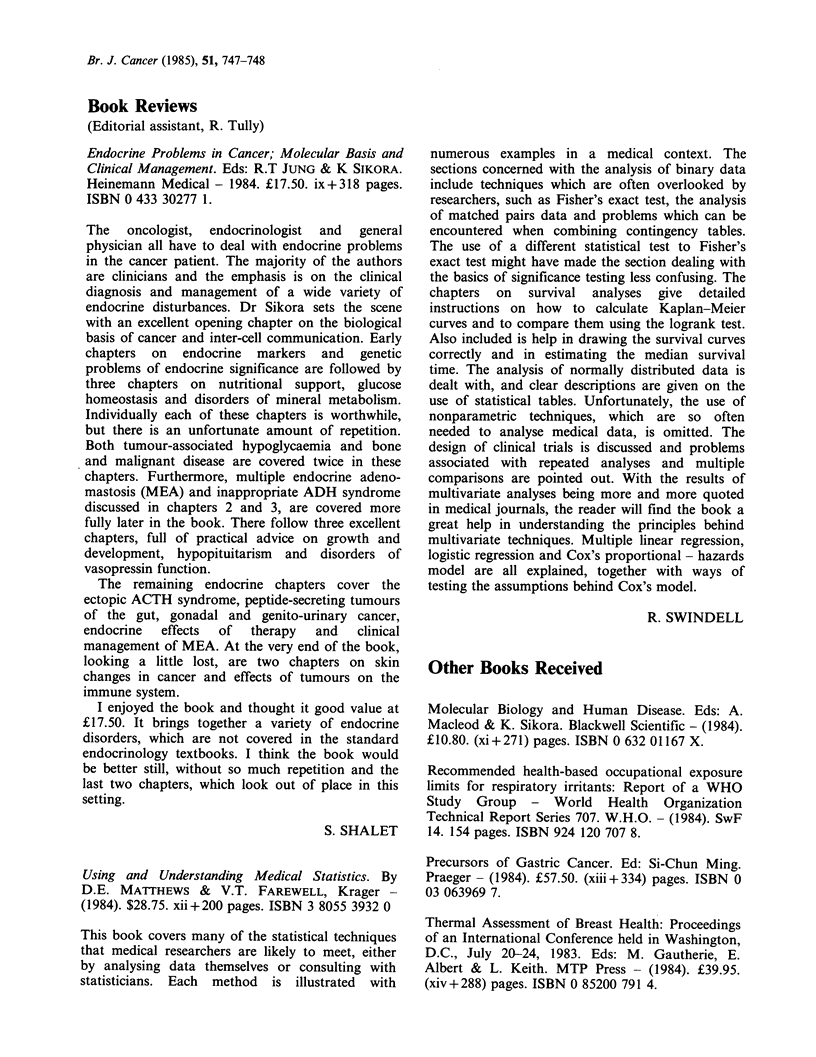# Endocrine Problems in Cancer; Molecular Basis and Clinical Management

**Published:** 1985-05

**Authors:** S. Shalet


*Br. J. Cancer* (1985), **51**, 747–748

### Book Reviews

(Editorial assistant, R. Tully)

*Endocrine Problems in Cancer; Molecular Basis and Clinical Management*. Eds: R.T JUNG & K SIKORA. Heinemann Medical – 1984. £17.50. ix+318 pages. ISBN 0 433 30277 1.

The oncologist, endocrinologist and general physician all have to deal with endocrine problems in the cancer patient. The majority of the authors are clinicians and the emphasis is on the clinical diagnosis and management of a wide variety of endocrine disturbances. Dr Sikora sets the scene with an excellent opening chapter on the biological basis of cancer and inter-cell communication. Early chapters on endocrine markers and genetic problems of endocrine significance are followed by three chapters on nutritional support, glucose homeostasis and disorders of mineral metabolism. Individually each of these chapters is worthwhile, but there is an unfortunate amount of repetition. Both tumour-associated hypoglycaemia and bone and malignant disease are covered twice in these chapters. Furthermore, multiple endocrine adenomastosis (MEA) and inappropriate ADH syndrome discussed in chapters 2 and 3, are covered more fully later in the book. There follow three excellent chapters, full of practical advice on growth and development, hypopituitarism and disorders of vasopressin function.

The remaining endocrine chapters cover the ectopic ACTH syndrome, peptide-secreting tumours of the gut, gonadal and genito-urinary cancer, endocrine effects of therapy and clinical management of MEA. At the very end of the book, looking a little lost, are two chapters on skin changes in cancer and effects of tumours on the immune system.

I enjoyed the book and thought it good value at £17.50. It brings together a variety of endocrine disorders, which are not covered in the standard endocrinology textbooks. I think the book would be better still, without so much repetition and the last two chapters, which look out of place in this setting.

S. SHALET